# Association of serum homocysteine with vitamin B12 and folate levels in women with pre-eclampsia in a tertiary health care center in Nepal

**DOI:** 10.1186/s12905-024-03284-9

**Published:** 2024-08-09

**Authors:** Binod Kumar Yadav, Suvana Maskey, Aseem Bhattarai, Salina Pradhananga, Sabina Shakya, Astha Regmi

**Affiliations:** 1 Institute of Medicine, Tribhuvan University Teaching Hospital, Maharajgunj, Kathmandu, Nepal; 2 Kantipur Dental College Teaching Hospital and Research Center, Kathmandu, Nepal; 3 Kathmandu Path Lab and Diagnostic Center, Kathmandu, Nepal; 4 Matrisishu Miteri Hospital, Pokhara, Nepal

**Keywords:** Pre-eclampsia, Hypertension, Homocysteine, Pregnancy

## Abstract

**Background:**

Pre-eclampsia is a syndrome that chiefly includes the development of new-onset hypertension and proteinuria after 20 weeks of pregnancy. Pre-eclampsia is one of the major causes of mortality and morbidity in Nepal. Hyperhomocysteinemia may be a cause of the endothelial dysfunction provoked by oxidative stress in pre-eclampsia. This study was designed to evaluate the association of homocysteine with Vitamin B12 and folate in patients with pre-eclampsia.

**Method:**

An observational cross sectional study was performed in the Gynecology and Obstetrics Department of TUTH involving seventy two subjects with pre-eclampsia. Blood pressure, urinary protein levels, serum homocysteine, Vitamin B12 and folate levels were compared in both mild and severe forms of pre-eclampsia. Concentration of Vitamin B12 and folate were measured using Vitros ECI and homocysteine was measured using CLIA. SPSS 23.0 was used to analyze the data. Tests were performed with Mann Whitney Test and Spearman’s rank correlation test. A p-value < 0.05 was considered statistically significant.

**Results:**

This study showed no significant difference in age and weeks of gestation in both mild and severe forms of pre-eclampsia. Mean concentration of homocysteine was higher (13.1 ± 6.4 micromol/L) in severe Pre-eclampsia as compared to mild cases (7.6 ± 2.8 micromol/L). Mean concentration of folate was lower in severe cases (35.4 ± 24.1 micromol/L) when compared with mild cases of pre-eclampsia (57 ± 23.4 micromol/L).

**Conclusion:**

Homocysteine levels were increased in severe Pre-eclampsia when compared with mild pre-eclampsia and this finding can be used to predict and prevent complications in patients with pre-eclampsia.

## Introduction

Pre-eclampsia is considered as the new onset of hypertension and proteinuria after 20 weeks of gestation in a previously normotensive patient [1–3]. Pre-eclampsia affects approximately 2–8% of pregnancies worldwide [4]. The World Health Organization (WHO) estimated that the incidence rate of preeclampsia in developing countries is 7-times higher (2.8%) than in developed countries (0.45%) [5]. In Nepal, the incidence of preeclampsia or eclampsia was 20 cases to 1,000 hospital deliveries [6].

Both maternal and fetal/placental variables are probably involved in the pathophysiology of pre-eclampsia. This systemic condition is closely linked to pathological placental blood flow, endothelial activation, oxidative stress and generalized inflammation. The most prominent feature of this disease is endothelial dysfunction, which is caused by the release of soluble Fms-like tyrosine kinase 1(sFlt1), soluble endoglin and low levels of proangiogenic factors like placental growth factor (PlGF), vascular endothelial growth factor (VEGF) from the ischemic placenta into maternal plasma [7]. Poor uterine and placental perfusion as a result of improper implantation and placentation causes oxidative stress, hypoxia, and the production of several anti-angiogenic factors, all of which contribute to generalised endothelial dysfunction [8].

Micronutrients like folate and Vitamin B12, which are components of the one carbon cycle are important elements of pregnancy outcome. It has been recommended that the pathophysiology of pre-eclampsia may be influenced by an imbalance in the levels of folate and other B vitamins [9, 10]. It is well known that low levels of folate and B12 can lead to increased levels of homocysteine, which further result in increased oxidative stress [11]. Tetrahydofolate, the active form of folate, can only be produced through the conversion of folate with the help of Vitamin B12. An impaired folate and vitamin B12 status is known to alter homocysteine levels [12]. Folate deficiency could be one of such factors that affects the vasculogenesis of the yolk sac, embryonic tissues and placenta. Trophoblast apoptosis is a possible mechanism because of hyperhomocysteinemia during early pregnancy prior to the development of pre-eclampsia [13].

Raised levels of homocysteine have been linked with atherosclerosis and cardiovascular disorders attributing to endothelial dysfunction [14]. Maternal plasma homocysteine concentration has been proposed to be associated with pregnancy complications including pre-eclampsia, placental abruption, intrauterine growth restriction (IUGR), and pregnancy loss [15]. Hyperhomocysteinemia has been hypothesized to be associated with preeclampsia because of its role in endothelial dysfunction [16]. Although some of the previous reports suggested several possible mechanisms for the occurrence of hyperhomocysteinemia in pre-eclampsia, the exact mechanisms are not known. The purpose of present study is to find the association of homocysteine, folate and B12 levels in women diagnosed with preeclampsia.

## Materials and methods

This study was carried out at Institute of Medicine, Tribhuvan University Teaching Hospital, Maharajgunj, Kathmandu. Institutional Review Board at the Tribhuvan University, Institute of Medicine, Maharajgunj, Nepal approved the study (Approval number: 368 [6–11]E^2^ /075 /76). All the participants completed a medical history form and provided informed consent. Seventy two Pre-eclamptic patients in the age group 19–44 years were studied for estimation of serum homocysteine, folic acid and vitamin B12 over a period of February 2019 to November 2019.

Pre-eclampsia is considered as the onset of a new episode of hypertension during pregnancy (with persistent systolic blood pressure > 140 mm Hg and diastolic blood pressure > 90 mm Hg) with the occurrence of substantial proteinuria (> 0.3 g/24 h). The clinical findings of Preeclampsia are assorted by the presence of systemic endothelial dysfunction and microangiopathy, affecting the liver (hemolysis, elevated liver function tests and low platelet count, namely HELLP syndrome) and the kidney (proteinuria) [8].

Patients with symptoms and signs suggestive of eclampsia supported by laboratory investigations and with normal renal and liver function tests were included in the study. However, patients were excluded from the study in case of multiple gestation, placental insufficiency, seizure disorder, history of chronic hypertension, causes of Proteinuria other than Pre-eclampsia, history of cardiovascular disease and in-vitro fertility related pregnancies.

In all patients, blood samples were obtained by taking aseptic precautions and then placed in serum separator tubes. Blood samples were centrifuged at 4,000 rpm for 5 min at room temperature and the separated serum was stored at -40 °C until the assays were performed.

The concentration of serum Vitamin B12 and folate levels were assayed by immunochemiluminescence technique by using VITROS 3600 (Ortho Clinical Diagnostics). Measuring range for Vitamin B12 is 50-2000 pg/ml and measuring range for folate is 0.6–20 ng/ml. The serum homocysteine level was measured by competitive binding immunoassay technique by using ADVIA Centaur CP (Siemens Healthcare Diagnostics). Measuring range for homocysteine is 1–50 micromol/L.

Data analyses included standard descriptive statistics using SPSS version 23 (IBM Corporation, Armonk, NY, USA), with variables expressed as mean ± standard deviation or medians as appropriate. Tests were performed with Mann Whitney Test and Spearman’s rank correlation test. A P-value of < 0.05 was considered statistically significant.

## Results

The mean age of patients diagnosed with Pre-eclampsia was 28.2 ± 5.1 years. The mean gestational age of the patients diagnosed with Pre-eclampsia was 35.5 ± 3.7 WOG (weeks of gestation). (Table 1) Forty (55%) women with Pre-eclampsia were Primigravid and thirty two (45%) women were multigravida.

**Table 1:** Descriptive characteristics for various parameters in the study levels

Parameters	Mean	Median (IQR)	95% CI
Age (years)	28.2 ± 5.1	27.5 (7)	(25,29)
Gestational age (WOG)	35.5 ± 3.7	36.0 (6)	(35, 38)
DBP(mm Hg)	100.9 ± 8.7	100.0 (20)	(100, 100)
SBP(mm Hg)	148.6 ± 9.9	150.0 (20)	(140, 150)
Body Mass Index (kg/m^2^)	30.5 ± 4.1	30.10 (6.6)	(28.6, 32.03)
Homocysteine(micromol/L)	9.57 ± 5 0.10	8.2 (5.2)	(7.3, 9.8)
Vitamin B12(pg/ml)	213.2 ± 62.0	201.0 (85.8)	(191, 223)
Folate(ng/ml)	49.53 ± 25.7	53.5(40.5)	(36, 63)

Cases of Pre-eclampsia were divided into mild and severe cases as defined by the American College of Obstetricians and Gynecologists 2012–2013 [17]. Forty-seven (65%) cases with preeclampsia were mild and Twenty five (35%) cases with pre-eclampsia were severe.

Mean levels of serum homocysteine in mild and severe cases of Pre-eclampsia were 7.6 ± 2.8 micromol/L and 13.1 ± 6.4 micromol/L respectively. Mean serum Vitamin B12 levels of women with mild and severe Pre-eclampsia were 220 ± 61.7pg/ml and 200 ± 61.7 pg/ml respectively. Mean serum folate levels of women with mild and severe pre-eclampsia were 57 ± 23.4 ng/ml and 35 ± 24.1 ng/ml respectively. (Table 2)

**Table 2:** Descriptive characteristics for various parameters in patients with pre-eclampsia

Parameters	Mild	Severe
Age (years)	28.1 ± 4.5	28.2 ± 6.2
Gestational age (WOG)	36.3 ± 3	34.0 ± 4.4
Body Mass Index (kg/m^2^)	30.6 ± 3.8	30.2 ± 4.9
Homocysteine (micromol/L)	7.6 ± 2.8	13.1 ± 6.4
Vitamin B12 (pg/ml)	220 ± 61.7	200.5 ± 61.7
Folate (ng/ml)	57 ± 23.4	35.4 ± 24.1

The Mann Whitney results indicated that homocysteine levels in severe pre-eclampsia was significantly higher than in mild pre-eclampsia. (Z value: -4.15, *p* = 0.001) and folate levels were low in severe Pre-eclampsia than in mild pre-eclampsia. (Z value: − 3.36, *p* = 0.001) (Table 3).

**Table 3:** Mann Whitney Test results for categories of pre-eclampsia

Variable	Group	Mean Rank	Z	Sig (2-tailed
Homocysteine	**Mild**	29.0	-4.15	0.000*
	**Severe**	50.5		
Vitamin B12	**Mild**	39.4	-1.62	0.104
	**Severe**	31.0		
Folate	**Mild**	42.5	-3.36	0.001*
	**Severe**	25.1		

*Significant at the 0.01 level

A spearman’s correlation was done to find out the correlation between homocysteine, folate and B12 according to severity of Pre-eclampsia. There was significant correlation found between homocysteine and folate levels (*p* value 0.001) and homocysteine and B12 levels (*p* value 0.002). (Table 4)

**Table 4:** Correlation of means of homocysteine, vitamin B12 and folate

Spearman‘s Rho	Homocysteine	B12	Folate
Homocysteine Correlation coefficient	-	0.365	0.647
Sig. (2 tailed)	-	0.002*	0.001*

*Significant at the 0.05 level

## Discussion

The present study tested the levels of maternal plasma folate, vitamin B12 and homocysteine in women with pre-eclampsia. Our outcomes demonstrated a few remarkable observations in Pre-eclampsia which are as follows: (1) Serum levels of homocysteine were raised in subjects with severe pre-eclampsia contrasted to mild pre-eclampsia. (2) Serum levels of folate were decreased in severe pre-eclampsia contrasted to mild pre-eclampsia. (3) Homocysteine and vitamin B12 and homocysteine and folate showed a strong link (p value < 0.01), whereas folate and vitamin B12 showed no significant association.

In this study, the mean homocysteine was found to be 9.57 micromol/L. Similar study done by Rajkovic et al. reported a mean homocysteine level of 12.7 micromol/L as compared to 9.93 micromol/L in normal pregnant women, suggesting association between homocysteine levels and pre-eclampsia [18]. The data of the present study is consistent with the previous studies of Bergen et al [19] and Braekke et al [20] who also made discoveries of raised levels of homocysteine in Pre-eclampsia. In normal pregnancy, the plasma concentration of homocysteine tends to decrease. This may be because of increased plasma volume and resulting hemodilution, increased glomerular filtration rate, pregnancy related hormonal changes and increased uptake of homocysteine by the fetus [21–23]. It is believed that increased homocysteine in pre-eclampsia damages the vascular endothelium. Furthermore, the endothelium in pregnant women may be more predisposed to harm, even a little rise in homocysteine levels has the potential to damage the endothelium and activate a number of mechanisms that cause Pre-eclampsia.

Folate, Vitamin B12 and B6 are necessary for DNA synthesis and cell proliferation and are involved in homocysteine metabolism. In the present study, we found that severe pre-eclampsia had lower levels of folate than moderate pre-eclampsia. Some observational studies that show reduced levels of folate in pregnant women with pre-eclampsia confirm our findings [19, 24–27]. In contrast, few studies report higher levels of folate [28–30]. In a systematic review by Mignini et al., folic acid and vitamin B12 concentrations were lower in preeclamptic women when compared with those of normotensive women [31]. The results of this study are in line with a number of studies conducted by Yanez et al. and Mujawar et al., who reported similar relationships between higher homocysteine concentration and lower folate concentrations [24, 32]. Homocysteine is metabolized by pathways: remethylation and transsulfuration. Vitamin B6 is required for the transulfuration of homocysteine to cysteine while, folic acid has to be present as 5-methyl-tetrahydrofolate (FMTHF) to be an effective donor for the remethylation of homocysteine to methionine [33]. Homocysteine builds up as a result of impaired homocysteine re-methylation to methionine caused by insufficient vitamin B availability (B_6_, B_12_ and folic acid) [34].

In summary, homocysteine level is still a relatively novel term in obstetrics, especially in a developing country like Nepal. This must be the first study done in Nepalese population that showed increased levels of homocysteine and decreased levels of folate in severe form of pre-eclampsia as compared to mild pre-eclampsia. Although majority of women had decreased Vitamin B12 levels, there was no significant difference when mild and severe forms of preeclampsia were compared. Though higher homocysteine levels have been shown to be associated with pre-eclampsia and may also be considered as a predictive risk factor for preeclampsia and yet, it cannot be used as a screening tool.

Our finding is important for future clinical studies in suspected cases of pre-eclampsia. The limitations of our study is that the samples were exclusively obtained from suspected cases of pre-eclampsia. The inclusion of case-control studies would have strengthened the study to establish association between homocysteine, vitamin B12 and folate levels. Additionally, we recognized that not following up with patients with pre-eclampsia until delivery limits the depth of our statistical analysis regarding homocysteine levels and their feto-maternal outcomes.

## Conclusion

From the above discussion, we can assume that biochemical screening such as homocysteine, folic acid and vitamin B12 are of supreme importance in pre-eclampsia. Homocysteine levels were elevated in patients with severe Pre-eclampsia as compared to mild pre-eclampsia. Thus, hyperhomocysteinemia is a treatable risk factor as folic acid and vitamin B12 supplementation can help decrease plasma homocysteine concentration, thereby, reducing the risk of developing pre-eclampsia and its consequences. Conversely, further prospective, large scale, longitudinal studies is essential to define the practicality of homocysteine in evaluating pre-eclampsia.

## Data Availability

All data that are generated during this study are included in this article. The data that support the findings of this study are available on request from the corresponding author, Pradhananga et al., upon reasonable request. The data is not available due to the information that could compromise the privacy of the research participants.

## References

[1] Gestational Hypertension and Preeclampsia. ACOG Practice Bulletin, Number 222. Obstet Gynecol. 2020;135(6):e237–60. 32443079 10.1097/AOG.0000000000003891

[2] Payne B, Magee LA, von Dadelszen P. Assessment, surveillance and prognosis in pre-eclampsia. Best Pract Res Clin Obstet Gynaecol. 2011;25(4):449–62. 21459048 10.1016/j.bpobgyn.2011.02.003

[3] Visintin C, Mugglestone MA, Almerie MQ, Nherera LM, James D, Walkinshaw S, et al. Management of hypertensive disorders during pregnancy: summary of NICE guidance. BMJ. 2010;341:c2207. 20739360 10.1136/bmj.c2207

[4] Roberts JM, Pearson GD, Cutler JA, Lindheimer MD, National Heart L, Blood I. Summary of the NHLBI working group on research on hypertension during pregnancy. Hypertens Pregnancy. 2003;22(2):109–27. 12908996 10.1081/PRG-120016792

[5] Osungbade KO, Ige OK. Public health perspectives of preeclampsia in developing countries: implication for health system strengthening. J Pregnancy. 2011;2011:481095. 21547090 10.1155/2011/481095 PMC3087154

[6] Manandhar BL, Geater CV. Antenatal Care and severe pre-eclampsia in Kathmandu valley. J Chitwan Med Coll. 2013;3(6):43–7.

[7] Eiland E, Nzerue C, Faulkner M. Preeclampsia 2012. J Pregnancy. 2012;2012:586578. 22848831 10.1155/2012/586578 PMC3403177

[8] Al-Jameil N, Aziz Khan F, Fareed Khan M, Tabassum H. A brief overview of preeclampsia. J Clin Med Res. 2014;6(1):1–7. 24400024 10.4021/jocmr1682w PMC3881982

[9] Kharb S, Aggarwal D, Bala J, Nanda S. Evaluation of homocysteine, vitamin B12 and folic acid levels during all the trimesters in pregnant and Preeclamptic Womens. Curr Hypertens Rev. 2016;12(3):234–8. 27748186 10.2174/1573402112666161010151632

[10] Acilmis YG, Dikensoy E, Kutlar AI, Balat O, Cebesoy FB, Ozturk E, et al. Homocysteine, folic acid and vitamin B12 levels in maternal and umbilical cord plasma and homocysteine levels in placenta in pregnant women with pre-eclampsia. J Obstet Gynaecol Res. 2011;37(1):45–50. 21040211 10.1111/j.1447-0756.2010.01317.x

[11] Fenech M. Folate (vitamin B9) and vitamin B12 and their function in the maintenance of nuclear and mitochondrial genome integrity. Mutat Res. 2012;733(1–2):21–33. 22093367 10.1016/j.mrfmmm.2011.11.003

[12] Wadhwani NS, Patil VV, Mehendale SS, Wagh GN, Gupte SA, Joshi SR. Increased homocysteine levels exist in women with preeclampsia from early pregnancy. J Matern Fetal Neonatal Med. 2016;29(16):2719–25. 26552939 10.3109/14767058.2015.1102880

[13] Steegers-Theunissen RP, Steegers EA. Nutrient-gene interactions in early pregnancy: a vascular hypothesis. Eur J Obstet Gynecol Reprod Biol. 2003;106(2):115–7. 12551774 10.1016/S0301-2115(02)00358-5

[14] Shih CC, Shih YL, Chen JY. The association between homocysteine levels and cardiovascular disease risk among middle-aged and elderly adults in Taiwan. BMC Cardiovasc Disord. 2021;21(1):191. 33879044 10.1186/s12872-021-02000-x PMC8056530

[15] Dodds L, Fell DB, Dooley KC, Armson BA, Allen AC, Nassar BA, et al. Effect of homocysteine concentration in early pregnancy on gestational hypertensive disorders and other pregnancy outcomes. Clin Chem. 2008;54(2):326–34. 18070815 10.1373/clinchem.2007.097469

[16] Chaudhry SH, Taljaard M, MacFarlane AJ, Gaudet LM, Smith GN, Rodger M, et al. The role of maternal homocysteine concentration in placenta-mediated complications: findings from the Ottawa and Kingston birth cohort. BMC Pregnancy Childbirth. 2019;19(1):75. 30782144 10.1186/s12884-019-2219-5 PMC6381683

[17] Gynecologists ACoOa. Hypertension in pregnancy. Report of the American College of Obstetricians and gynecologists’ task force on hypertension in pregnancy. Obstet Gynecol. 2013;122(5):1122. 24150027 10.1097/01.AOG.0000437382.03963.88

[18] Rajkovic A, Mahomed K, Malinow MR, Sorenson TK, Woelk GB, Williams MA. Plasma homocyst(e)ine concentrations in eclamptic and preeclamptic African women postpartum. Obstet Gynecol. 1999;94(3):355–60. 10472859 10.1016/s0029-7844(99)00304-x

[19] Bergen NE, Jaddoe VW, Timmermans S, Hofman A, Lindemans J, Russcher H, et al. Homocysteine and folate concentrations in early pregnancy and the risk of adverse pregnancy outcomes: the Generation R Study. BJOG. 2012;119(6):739–51. 22489763 10.1111/j.1471-0528.2012.03321.x

[20] Braekke K, Ueland PM, Harsem NK, Karlsen A, Blomhoff R, Staff AC. Homocysteine, cysteine, and related metabolites in maternal and fetal plasma in preeclampsia. Pediatr Res. 2007;62(3):319–24. 17622947 10.1203/PDR.0b013e318123fba2

[21] Hague WM. Homocysteine and pregnancy. Best Pract Res Clin Obstet Gynaecol. 2003;17(3):459–69. 12787538 10.1016/S1521-6934(03)00009-9

[22] Herrmann W, Hubner U, Koch I, Obeid R, Retzke U, Geisel J. Alteration of homocysteine catabolism in pre-eclampsia, HELLP syndrome and placental insufficiency. Clin Chem Lab Med. 2004;42(10):1109–16. 15552268 10.1515/CCLM.2004.228

[23] Wang J, Trudinger BJ, Duarte N, Wilcken DE, Wang XL. Elevated circulating homocyst(e)ine levels in placental vascular disease and associated pre-eclampsia. BJOG. 2000;107(7):935–8. 10901568 10.1111/j.1471-0528.2000.tb11095.x

[24] Mujawar SA, Patil VW, Daver RG. Study of serum homocysteine, folic acid and vitamin b(12) in patients with preeclampsia. Indian J Clin Biochem. 2011;26(3):257–60. 22754189 10.1007/s12291-011-0109-3 PMC3162959

[25] Salehi-Pourmehr H, Mohamad-Alizadeh S, Malakouti J, Farshbaf-Khalili A. Association of the folic acid consumption and its serum levels with preeclampsia in pregnant women. Iran J Nurs Midwifery Res. 2012;17(6):461–6. 23922590 PMC3733294

[26] Patrick TE, Powers RW, Daftary AR, Ness RB, Roberts JM. Homocysteine and folic acid are inversely related in black women with preeclampsia. Hypertension. 2004;43(6):1279–82. 15096466 10.1161/01.HYP.0000126580.81230.da

[27] Sanchez SE, Zhang C, Rene Malinow M, Ware-Jauregui S, Larrabure G, Williams MA. Plasma folate, vitamin B(12), and homocyst(e)ine concentrations in preeclamptic and normotensive Peruvian women. Am J Epidemiol. 2001;153(5):474–80. 11226979 10.1093/aje/153.5.474

[28] Also-Rallo E, Lopez-Quesada E, Urreizti R, Vilaseca MA, Lailla JM, Balcells S, et al. Polymorphisms of genes involved in homocysteine metabolism in preeclampsia and in uncomplicated pregnancies. Eur J Obstet Gynecol Reprod Biol. 2005;120(1):45–52. 15866085 10.1016/j.ejogrb.2004.08.008

[29] Lopez-Quesada E, Vilaseca MA, Lailla JM. Plasma total homocysteine in uncomplicated pregnancy and in preeclampsia. Eur J Obstet Gynecol Reprod Biol. 2003;108(1):45–9. 12694969 10.1016/S0301-2115(02)00367-6

[30] Powers RW, Dunbar MS, Gallaher MJ, Roberts JM. The 677 C-T methylenetetrahydrofolate reductase mutation does not predict increased maternal homocysteine during pregnancy. Obstet Gynecol. 2003;101(4):762–6. 12681883 10.1016/s0029-7844(02)03120-4

[31] Mignini LE, Latthe PM, Villar J, Kilby MD, Carroli G, Khan KS. Mapping the theories of preeclampsia: the role of homocysteine. Obstet Gynecol. 2005;105(2):411–25. 15684173 10.1097/01.AOG.0000151117.52952.b6

[32] Yanez P, Vasquez CJ, Rodas L, Duran A, Chedraui P, Liem KH, et al. Erythrocyte folate content and serum folic acid and homocysteine levels in preeclamptic primigravidae teenagers living at high altitude. Arch Gynecol Obstet. 2013;288(5):1011–5. 23609037 10.1007/s00404-013-2851-7

[33] Finkelstein JD. The metabolism of homocysteine: pathways and regulation. Eur J Pediatr. 1998;157(Suppl 2):S40–4. 9587024 10.1007/PL00014300

[34] Mangge H, Becker K, Fuchs D, Gostner JM. Antioxidants, inflammation and cardiovascular disease. World J Cardiol. 2014;6(6):462–77. 24976919 10.4330/wjc.v6.i6.462 PMC4072837

